# Bridging sepsis physiology and biosensor engineering: a clinical perspective on electrochemical cytokine sensor design

**DOI:** 10.3389/fbioe.2026.1942504

**Published:** 2026-09-17

**Authors:** M. Walkowicz, J. Jarczewski, B. Słomiński, S. Bierzanowski, K. Pietruczuk, K. P. Steckiewicz

**Affiliations:** 1 Department of Medical Immunology, Faculty of Medicine, Medical University of Gdańsk, Gdańsk, Poland; 2 Division of Biostatistics and Neural Networks, Medical University of Gdańsk, Gdańsk, Poland; 3 Department of Anesthesiology and Intensive Therapy, Faculty of Medicine, Medical University of Gdańsk, Gdańsk, Poland; 4 Department of Anesthesiology and Intensive Therapy, University Clinical Centre, Gdańsk, Poland

**Keywords:** biosensor, cytokines, intensive care, point-of-care, sepsis

## Abstract

Sepsis remains one of the most critical challenges in contemporary intensive care medicine, characterized by profound immunological and physiological heterogeneity. Cytokines have gained increasing attention as dynamic biomarkers that can complement conventional markers and bedside clinical assessment, enabling more refined diagnosis, risk stratification, and monitoring of treatment response. Most current work on cytokine biosensing is organized around a technological ambition: continuous, minimally invasive, wearable measurement. In this Perspective, we invert that starting point and derive sensor specifications from the pathophysiology and workflow of the septic patient in intensive care. Doing so yields a different set of priorities. Because septic concentrations of the principal mediators span three to four orders of magnitude, a wide linear range matters more than the lowest attainable limit of detection. Because resuscitation and antimicrobial decisions are made within the first hours, response times must be measured in minutes rather than tens of minutes. Because reference ranges, kinetics and outcome associations have been established only in blood, and because vascular access is already present in these patients, whole blood remains the medium of choice, and a discrete, single-use format is preferable to continuous operation in a fouling-prone matrix. Several of these conclusions run counter to the direction the field is currently taking. We argue that aligning sensor architecture with the pathophysiological context of sepsis is a precondition for cytokine-targeted biosensing to support decision-making at the bedside.

## 1 Introduction

Sepsis is a life-threatening, multiorgan dysfunction caused by a dysregulated host immune response that remains one of the most formidable challenges in modern medicine. Currently, it accounts for approximately 20% of global deaths (Rudd et al., 2020), while septic shock can exhibit a 30%–40% mortality rate (La Via et al., 2024). The timing of intervention is septic shock therapy (Kumar et al., 2006); every hour of delay in treatment initiation is associated with a 4.1% increase in mortality risk (Tang et al., 2024b), yet a delay in the administration of the second antibiotic dose affects approximately 20% of patients (Randolph et al., 2021). Although sepsis remains a purely clinical diagnosis, laboratory parameters can support the diagnosis of sepsis by broadening the clinical context, especially in ambiguous cases and high-risk populations (Prescott et al., 2026). Cytokines can play a vital supportive role in the identification of sepsis and assessment of its severity (Matsumoto et al., 2018; Fajgenbaum and June, 2020; Goyal et al., 2024). However, there is an urgent clinical need for rapid, point-of-care measurements to capture the rapid dynamics of the immune response and enable personalized treatment strategies (Llitjos et al., 2024).

Electrochemical biosensors have gained significant attention as a solution to this need, offering rapid and precise quantification of cytokine concentrations across various biological fluids (Kim et al., 2021). At present, ELISA remains the gold standard for quantitative cytokine assessment; however, standard laboratory ELISA assays are relatively time-consuming, costly and typically limited to single-analyte measurements, which limits their suitability for rapid bedside diagnostics and for serial monitoring of inflammatory dynamics in septic patients (Antcliffe et al., 2025). However, translating these sensors from the laboratory to the bedside involves overcoming several critical engineering and clinical constraints, including detection range, operational stability, and cost-effectiveness. In this Perspective, we present our view on the key requirements that electrochemical biosensors must fulfil to effectively bridge the gap between engineering innovation and clinical utility, ultimately optimizing sepsis diagnosis and therapy monitoring.

## 2 Sepsis: A multifaceted adversary

The etiology and clinical presentation of sepsis exhibit profound heterogeneity among patients. A fundamental challenge in establishing an accurate diagnosis and initiating effective treatment lies in characterizing the specific phenotype (the predominant direction of the host’s immune response) and endotype (the distinct pathophysiological pathways leading to specific clinical manifestations) (Tang et al., 2024a; Antcliffe et al., 2025). The primary immune phenotypes are categorized as hyperinflammatory and immunosuppressive. An excessive immunological response characterizes the first one, while the latter involves a pathological suppression of immune competence (Santacroce et al., 2024). It is increasingly recognized that these two processes often overlap or occur concurrently, a state that significantly contributes to patient mortality (Ricaño-Ponce et al., 2022). While phenotype identification does not yet guide a single universally effective intervention, it enables clinicians to significantly narrow the diagnostic scope and tailor therapeutic interventions.

### 2.1 Cytokines: Orchestrators of sepsis pathogenesis and diagnostic milestones

To date, over 250 biomarkers have been identified for the diagnosis and phenotyping of sepsis (Pierrakos et al., 2020), though many remain purely auxiliary. Current Surviving Sepsis Campaign guidelines (Prescott et al., 2026) support the use of procalcitonin (PCT) primarily as a tool for antimicrobial stewardship. However, PCT is a relatively late marker; its concentration typically peaks around 24 h after onset, meaning that early testing may yield false-negative results (Chairaj et al., 2026). In contrast, a growing body of evidence highlights the superior prognostic value of cytokines (Li et al., 2023). As biomarkers, cytokines are particularly advantageous because they reflect immunological shifts far more rapidly than the current “gold standards” PCT and C-reactive protein (CRP) (Jekarl et al., 2019). Among the most clinically relevant cytokines in sepsis are TNF-α, IL-1β, and IL-6, which act as major pro-inflammatory mediators (Gharamti et al., 2022; Varga et al., 2025); IL-8, a key predictor of acute organ damage (Anderson et al., 2019); IL-10, primarily associated with the onset of immunosuppression (Zhou et al., 2024); and IP-10, useful for distinguishing bacterial from viral etiologies (Oved et al., 2015). The strategic use of cytokines facilitates the classification of patients into specific sepsis phenotypes (Pierrakos et al., 2020; Candelli et al., 2025). Multi-marker panels are proving especially valuable, such as the IL-6/IL-10 (Wang et al., 2026). This approach is also applicable to monitoring treatment efficacy; for instance, a decisive decrease in pro-inflammatory markers (e.g., TNF-α and IL-6) is strongly correlated with increased survival rates (Li et al., 2023). Consequently, it is difficult to reliably assess the intensity of the immune response based on a single measurement (Pierrakos et al., 2020). Furthermore, a point-in-time measurement provides incomplete information, as cytokine levels may be elevated due to non-septic comorbidities (Varga et al., 2025). Therefore, it is imperative to conduct serial measurements to accurately map the kinetics of cytokine fluctuations (Matsumoto et al., 2018), as this data constitutes an independent predictor of survival (Weidhase et al., 2019; Varga et al., 2025). General concentration ranges for healthy individuals versus septic patients are summarized in Table 1.

**TABLE 1:** Summary of the most clinically relevant cytokines in sepsis, including their physiological and critical concentrations.

Cytokine	Physiological levels (pg/ml)	Septic levels (pg/ml)	Role	Span
IL-6	2.18–2.27 (Yang et al., 2026)	164–8750/267-34,758 (Ruiz-Rodríguez et al., 2026); 45.2–272.6/256.4–11,062.1 (Song et al., 2019)	An early-rising pro-inflammatory cytokine whose plasma levels closely reflect sepsis onset and severity, making it a key biomarker for early diagnosis and risk stratification. Spanning more than four orders of magnitude from health to severe sepsis, IL-6 sets the dynamic-range requirement for any shared-architecture panel; sub-pg/mL sensitivity confers no additional clinical information	∼4.2 orders
IL-8	3.51–3.98 (Yang et al., 2026)	190–850 (Yousef et al., 2014); 85–588 (Madushani et al., 2022)	A potent neutrophil chemoattractant and a critical biomarker for predicting and monitoring sepsis-associated acute organ dysfunction and disease severity. Septic concentrations lie two orders above the physiological baseline, comfortably above the detection limit of most reported platforms, so linearity across the upper range rather than sensitivity is limiting	∼2.4 orders
IL-10	1.81–2.02 (Yang et al., 2026)	21–136 (Riché et al., 2013); 65–312/104–805 (Yan et al., 2025)	A potent anti-inflammatory cytokine whose elevated levels in sepsis are strongly associated with immunosuppression, organ failure and increased mortality. Reported septic ranges diverge substantially between studies, so a device intended to support thresholds must be calibrated against a defined comparator rather than to an assumed absolute range	∼2.7 orders
IP-10	∼150–200 (Madhurantakam et al., 2023)	1119–4342 (Ng et al., 2007)	A key biomarker of sepsis progression and a modulator of antibacterial immune responses. Because the physiological baseline already approaches 200 pg/mL, a limit of detection in the low pg/mL range is of no clinical benefit; the requirement is linearity to the upper septic values	∼1.5 orders
TNF-α	3.8–8 (Gharamti et al., 2022)	39.8–85.8 (Gharamti et al., 2022)	A central early mediator of the septic inflammatory response; alterations in its circulating levels or genetic regulation have been linked to sepsis susceptibility and outcome. Healthy and septic values are separated by little more than one order of magnitude at low absolute concentrations, so precision and limit of detection, rather than dynamic range, determine clinical utility	∼1.4 orders
IL-1β	1.17–1.39 (Yang et al., 2026)	13.4–91.6 (Gharamti et al., 2022)	A primary upstream orchestrator in sepsis, triggering the secretion of other pro-inflammatory cytokines such as TNF-α and IL-6. The lower septic bound lies near 13 pg/mL, which sets a practical ceiling of roughly 5 pg/mL on the acceptable limit of detection for this analyte	∼1.9 orders

Values are reported as concentration ranges (pg/ml). Because of methodological differences in cytokine quantification, reported concentrations vary between sources. Where a study distinguished between sepsis and septic shock, the combined range for both categories is given; where survivors and non-survivors were analyzed separately, the corresponding concentrations are separated by a slash. For meta-analyses, a single representative source was cited.

*IP-10:* values established in pediatric patients were adopted owing to the paucity of studies in adult populations (Ng et al., 2007); for comparison, the mean blood concentration of IP-10 in COVID-19, patients exceeded 1000 pg/ml (Madhurantakam et al., 2023).

Span denotes the approximate number of orders of magnitude separating the lowest reported physiological value from the highest reported septic value, and is given as an indication of the dynamic range a device must cover. The requirement is analyte-dependent: IL-6, and, to a lesser extent, IL-10, drive the dynamic-range requirement, whereas TNF-α and IL-1β separate healthy from septic values within a single order of magnitude at low absolute concentrations, where precision rather than range becomes limiting. A multiplexed device built on a shared architecture must therefore be dimensioned by its most demanding channel. The divergence between the septic ranges reported for IL-10 illustrates a wider difficulty: absolute thresholds derived from one cohort and assay do not transfer directly to another.

It must be borne in mind that patients differ in the balance of pro- and anti-inflammatory signaling, the pathogen driving the response, and in the temporal phase at which they present, so no single cytokine reliably identifies the condition across this range. For cytokines, moreover, no dedicated guidelines specify when a measurement should be obtained or what concentrations should be targeted - recommendations of this kind exist for lactate and, in the context of antimicrobial discontinuation, for procalcitonin (Prescott et al., 2026). Serial cytokine measurements also cannot replace the basic markers obtained in sepsis or septic shock, such as lactate, creatinine or bilirubin; unlike cytokines, these form the basis of the organ dysfunction and shock criteria that define the syndrome itself (Singer et al., 2016). Nor should they displace PCT or CRP: their kinetics are slower, but much of contemporary clinical practice rests on their interpretation (Chairaj et al., 2026). Because no single mediator captures this heterogeneity, multiplexed measurement is not a refinement but a requirement, and it has already been demonstrated for host-response panels in whole blood (Tanak et al., 2022). In practice, panels extending beyond two or three cytokines have rarely been evaluated, and the analytical behavior of larger arrays in a shared matrix remains largely uncharacterized. This shifts part of the analytical burden from the individual channel to the array: cross-reactivity between recognition elements, channel-to-channel calibration, and channel consistency within a shared matrix become limiting factors and are poorly characterized for cytokine panels at present.

### 2.2 The impact of sepsis on potential biosensing media

Sepsis profoundly alters systemic physiology, which is a critical consideration when selecting the optimal biological fluid for biosensor analysis. One of the earliest systemic signs of the inflammatory response is thermoregulatory dysregulation. In septic patients, this can manifest as either hypothermia or hyperthermia, occurring independently of ambient environmental conditions (Thomas-Rüddel et al., 2021). As sepsis severity increases, a pronounced core-to-peripheral temperature gradient is often observed (Hariri et al., 2019). Furthermore, sepsis induces capillary leak syndrome, characterized by increased vascular permeability that allows plasma to escape from the bloodstream into the surrounding tissues (McMullan et al., 2024; Pfortmueller et al., 2024). This phenomenon leads to an increase in interstitial fluid volume and is closely associated with microcirculatory dysfunction. Progressing sepsis typically leads to a decrease in systemic vascular resistance (SVR) due to widespread vasodilation and microcirculatory dysfunction, resulting in impaired tissue perfusion despite preserved or even increased cardiac output (De Backer et al., 2014).

#### 2.2.1 Blood

While considered the gold standard for biomarker analysis, blood concentrations are heavily influenced by aggressive fluid resuscitation - a foundational treatment in sepsis management (Prescott et al., 2026). Fluid therapy can alter cytokine levels both through direct biological interactions and mechanical hemodilution (Arnold et al., 2010; Erdogan et al., 2021).

#### 2.2.2 Interstitial Fluid (ISF)

This medium is an increasingly popular target for wearable biosensors. However, due to the aforementioned capillary leak, more than three-quarters of the fluids administered intravenously to the patient may ultimately migrate into the ISF (Coen, 2023; Marx, 2003). Although studies suggest that cytokine concentrations in the blood and ISF eventually reach equilibrium during sepsis (Koskela et al., 2017) (an effect not observed in healthy individuals (Sjöbom et al., 2020)), the exact temporal dynamics of this process remain unknown. This kinetic uncertainty poses a fundamental challenge for ISF-based sepsis biosensors, as the temporal lag between blood and ISF cytokine changes remains unquantified and may vary with disease severity.

#### 2.2.3 Sweat

The utility of sweat as a sensing medium in sepsis has not yet been thoroughly investigated. It is reasonable to assume that peripheral hypoperfusion and core-to-peripheral temperature gradients may significantly limit sweat production. While cytokines are known to be present in sweat (Marques-Deak et al., 2006) and their levels are elevated in certain inflammatory diseases (Jagannath et al., 2021; Hirten et al., 2024), there is currently no established correlation or kinetic model relating sweat cytokine concentrations to those found in the bloodstream during sepsis.

### 2.3 What is currently the most optimal medium for cytokine sampling in the ICU?

The positive case for blood is worth stating directly. Blood is the compartment in which cytokine concentrations have actually been measured in septic patients, and therefore the only one for which reference ranges, kinetics and outcome associations are established. Vascular access is already present in these patients, making repeated sampling a matter of workflow rather than of additional intervention, and a sample is obtained within minutes, whereas ISF (via microdialysis catheters) or sweat (via dedicated patches) may take tens of minutes to collect.

Choosing blood does not, by itself, specify the sample. Whole blood preserves the point-of-care advantage, since centrifugation reintroduces precisely the laboratory step that a bedside device is meant to eliminate. The anticoagulant is not neutral either: cytokine recovery differs systematically between EDTA, heparin and citrate, and between serum and plasma (de Jager et al., 2009; Hennø et al., 2017). Pre-analytical timing matters as much as tube choice: leukocytes and platelets continue to release cytokines *ex vivo*, so serum concentrations exceed those in plasma, and delayed centrifugation inflates IL-6 enough to alter clinical interpretation (Gong et al., 2019). This argues for bedside measurement rather than against it: whole blood remains, on current evidence, the most effective medium for point-of-care cytokine quantification.

## 3 Biosensors: Key features

Biosensing is a rapidly evolving field. Recent advancements have both enhanced established technologies and introduced novel sensing paradigms. In this Perspective, our primary objective is to highlight the potential benefits of effective sepsis marker detection. Rather than providing an exhaustive technical review of sensor architecture, we focus on the practical attributes - biorecognition element, measurement mode, sampling medium, and analytical performance - that decisively influence clinical utility in sepsis management.

At the transduction level, electrochemical techniques - amperometry, voltammetry (differential pulse, DPV; square-wave, SWV) and electrochemical impedance spectroscopy (EIS) - remain central, combining high sensitivity with compatibility with miniaturized, point-of-care platforms. Binding of a cytokine to a selective electrode interface perturbs measurable electrical parameters - current, potential, interfacial impedance - in a predictable manner within the predefined dynamic range of the device (Dutta et al., 2021). The choice among these techniques is not neutral with respect to the requirements set out above. Pulsed voltammetric interrogation of a tethered redox reporter - the signal-on/signal-off architecture of electrochemical aptamer-based sensors - suppresses capacitive background and enables reagentless, single-step operation, at the cost of possible steric interference with binding and susceptibility to endogenous redox-active species (White et al., 2008; Leung et al., 2023). Sensitivity and dynamic range are further governed by the electrode substrate and its nanostructuring (screen-printed carbon, gold thin films, composites with gold nanoparticles, graphene or carbon nanotubes) (Munge et al., 2009; Duan et al., 2025). Faradaic impedimetric detection is highly sensitive for discrete measurements, but it typically relies on a redox probe dissolved in the sample, which is difficult to reconcile with reagentless continuous operation, and it lacks an internal reference: because any interfacial change alters the recorded impedance, nonspecific adsorption and drift may be misread as specific binding. In a matrix as fouling-prone as whole blood this is a substantial liability for continuous formats, whereas an endpoint measurement on a freshly prepared electrode within a defined incubation window is far less exposed to it (Pham et al., 2025). Dual-frequency and dual-reporter schemes permit partial drift correction but remain engineering workarounds rather than solutions to the underlying interfacial instability. Because these constraints follow from the pathophysiology outlined above, the recognition layer must be selected under them - the subject of the following section.

### 3.1 Biorecognition elements

The fundamental component of an electrochemical biosensor is its electrode, upon which biorecognition molecules are immobilized to selectively bind target cytokines, thereby generating a measurable electrical signal change (Kim et al., 2021). Key differences among the biorecognition elements are shown in Figure 1.

**FIGURE 1:**
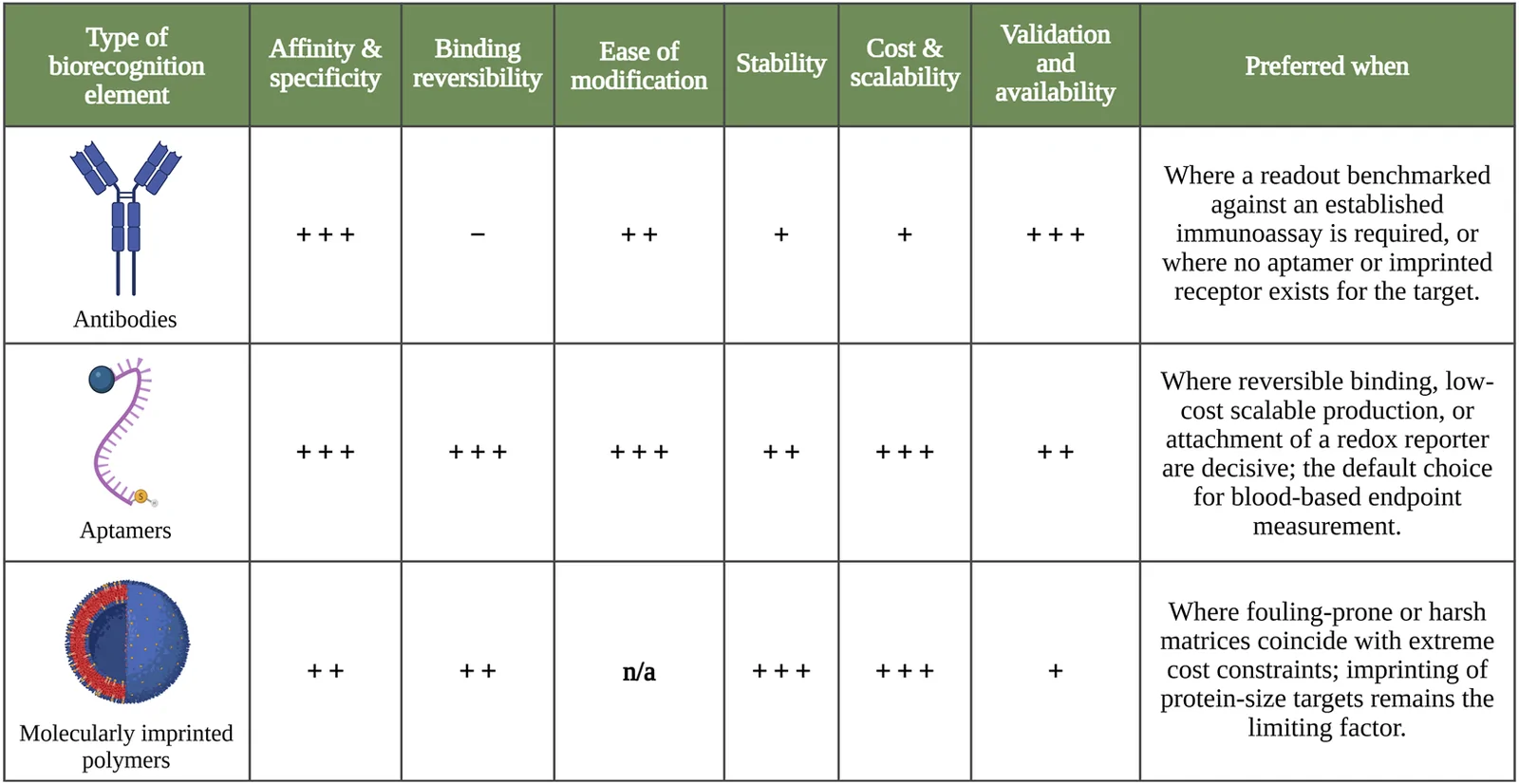
Comparison of biorecognition elements used in electrochemical cytokine biosensors. For each element, six attributes relevant to the requirements set out in Table 2 are rated, and the rightmost column indicates the conditions under which that element is the preferred choice. *Ease of modification* refers to the chemical functionalization of the recognition layer, including attachment of thiol groups and redox reporters; *validation and availability* refers jointly to the extent of clinical validation of a given class of receptor and to whether such a receptor exists for the cytokine of interest. Ratings reflect the properties discussed in Section 3.1 and are relative between the three classes rather than absolute. +++ favorable, ++ moderate, + limited, − unfavorable, n/a not applicable. Created in BioRender. Walkowicz, M. (2026) https://BioRender.com/d6c5bdp.

Antibodies represent the default choice for cytokine recognition, as these physiologically evolved proteins exhibit highly predictable binding behavior. Their principal advantages include exceptional specificity and resistance to non-specific interactions (Mpofu et al., 2025). However, this same characteristic can prove disadvantageous: antibody-cytokine binding is typically irreversible without destroying the electrode architecture, thereby limiting reusability.

Aptamers are short, single-stranded nucleic acid sequences (RNA or DNA) generated via SELEX (an iterative *in vitro* selection process in which large random nucleic acid libraries are enriched for sequences that bind a given target) that display specific affinity toward target proteins (Yang et al., 2023). Unlike antibodies, aptamers exhibit weaker, reversible binding, allowing bound cytokines to dissociate through fluid flow alone. They offer significant advantages in cost-effectiveness and chemical stability compared to antibodies (Reaño and Escobar, 2024; Li et al., 2025). However, limitations include incompletely characterized cross-interactions that may interfere with signal transduction in microneedle-based electrode arrays (Russell et al., 2019; Li et al., 2025), and the fact that aptamers have not yet been successfully synthesized for all clinically relevant cytokines.

Molecularly imprinted polymers (MIPs) represent an emerging alternative. These synthetic receptors function as molecular “molds” complementary to specific cytokine structures, offering notable stability and low production costs. Despite their economic appeal, MIPs face persistent challenges related to batch-to-batch reproducibility, template leakage, and performance in complex biological matrices (BelBruno, 2019).

### 3.2 Measurement mode and sampling medium

Biosensors are fundamentally categorized by their operational mode: single-use (point-of-care) versus continuous or near-real-time (RT/NRT) monitoring. Continuous measurement is primarily constrained by cumulative cytokine accumulation on the electrode surface. While certain antibody-based sensors achieve continuous operation through specialized electrode geometries (Song et al., 2021), aptamer-based sensors may be superior in this domain (Sánchez-Salcedo et al., 2023). Their relatively weak, reversible binding enables passive electrode regeneration through sample flow, yielding devices that maintain activity for up to 2 weeks *in vitro* and from 24 h to a few days in human volunteers (Jagannath et al., 2021; Hirten et al., 2024). Some NRT sensor designs employ an “active-reset” strategy, enabling measurements every 20–30 min concurrent with sensor regeneration (Zargartalebi et al., 2024). A critical challenge for continuous electrochemical monitoring is biofouling. It typically begins with rapid, nonspecific adsorption of plasma proteins (e.g., albumin, fibrinogen, immunoglobulins), Driven predominantly by hydrophobic and electrostatic interactions between the artificial surface and amphiphilic protein domains. This initial conditioning film masks the intrinsic surface chemistry, alters the electrical double layer, and impedes both electron transfer and analyte diffusion toward the sensor’s receptor layer. These processes reduce sensitivity and introduce baseline drift, shortening sensor lifetime in various environments (Liu et al., 2019). Although diverse protective coatings have been developed to mitigate nonspecific binding (Saxena et al., 2024), biofouling still restricts blood-based measurements predominantly to single-use, point-of-care formats, as blood contains the highest protein load and highly adhesive cellular components among common sampling media. Interstitial fluid (ISF) presents a somewhat more favorable environment for continuous biosensing due to lower protein and cellular content (Wu et al., 2024). However, sweat has so far emerged as the predominant medium for real-time or near-real-time cytokine monitoring (Ploner et al., 2024), despite the physiological limitations discussed in Section 2.2. Importantly, the technical feasibility of continuous monitoring does not automatically translate into clinical utility in sepsis.

### 3.3 Analytical performance metrics

The clinical utility of a biosensor is fundamentally determined by three key performance parameters: linear range - the concentration interval over which the sensor provides reliable quantification; limit of detection (LOD) - the smallest concentration change discernible above baseline noise; and response time - the interval from sample application to result generation. Current biosensor performance reveals significant limitations when benchmarked against sepsis requirements. A comprehensive survey of IL-6 sensors by McCrae et al. (2023) demonstrated that only 50% of reported devices achieved response times under 30 min, while fewer than 30% exhibited linear ranges exceeding the clinically relevant 10–500 pg/mL window. However, optimized designs demonstrate that balanced performance is achievable: sensors have been reported with LODs of 1 pg/mL, linear ranges spanning 1-10,000 pg/mL, and incubation times as brief as 5 min for detection in whole blood (Tanak et al., 2022). Critically, RT/NRT sensors typically require an initial equilibration period of several tens of minutes following deployment before the first measurement becomes available (Wu et al., 2024), a decisive limitation in sepsis.

## 4 Current requirements for cytokine biosensors

Cytokines are key mediators in sepsis. Growing attention is being paid to the fact that, to fully exploit their diagnostic potential, more effort must be devoted to implementing point-of-care tests or enabling continuous monitoring (Llitjos et al., 2024). Retrospective and clinical studies have also suggested that an inherently reduced sensitivity of the organism to selected cytokines may increase the chances of surviving sepsis (Leventogiannis et al., 2022; Linz et al., 2025; Giamarellos-Bourboulis et al., 2026). All these approaches, however, must be coupled to precise detection, which in this context may be even more important than the drug itself (Fan et al., 2025). During such detection, the main focus should not be placed on the absolute concentration of a single cytokine, but on the overall dynamics of its temporal changes (Abasıyanık et al., 2020; Llitjos et al., 2024; Samuelsen et al., 2024).

### 4.1 Illusions of perfection: Can contemporary biosensing models be applied in the context of sepsis?

At present, in the field of electrochemical biosensors, the greatest attention is given to concepts that propose continuous and minimally invasive cytokine monitoring, which are finding applications in certain chronic disease states or in monitoring the concentration of substances not directly related to acute pathologies. From the perspective of patient management in the intensive care unit, however, minimal invasiveness ceases to be a major advantage.

The design target depends on the clinical question being asked. Diagnosis requires discriminating infection-driven dysregulation from other causes of critical illness, favoring specificity and a decision threshold validated against an established comparator (Prescott et al., 2026). Risk stratification and phenotyping are concerned less with an absolute value than with the position of a patient within a distribution, which tolerates moderate analytical imprecision but demands consistency across sites and devices (Antcliffe et al., 2025). Monitoring of treatment response is the most demanding case: here the informative quantity is the direction and rate of change rather than any single value (Weidhase et al., 2019). This places the analytical burden on within-patient repeatability and on calibration stability across serial measurements. A single device is unlikely to serve all three equally, and the specifications summarized in Table 2 should therefore be read as application-dependent rather than absolute. Whatever role is assigned to a given cytokine measurement, however, at the present stage of sensor development it will most effectively be performed as a discrete, endpoint measurement on a sample of the patient’s blood.

**TABLE 2:** Target performance requirements for an electrochemical cytokine biosensor intended for use in sepsis.

Parameter	Target	Basis	Evidence
Linear range	≥3-4 orders of magnitude (≈1–10,000 pg/mL); the clinically informative quantity is the relative change between serial measurements rather than any absolute value	Septic concentrations span more than three orders of magnitude for the principal mediators (Table 1); hemodilution and capillary leak widen the range further (Marx, 2003); ranges of this width have been demonstrated on MWCNT-modified screen-printed electrodes (Liu et al., 2024)	Established
Limit of detection	≤5 pg/mL sufficient; lower preferable	Septic values rarely fall within the physiological range; the IP-10 baseline in health already exceeds 100 pg/mL (Ng et al., 2007), whereas TNF-α and IL-1β separate healthy from septic values at low absolute concentrations and therefore set the requirement (Gharamti et al., 2022)	Derived
Response time	<10 min from sample to result	Resuscitation and antimicrobial timelines (Prescott et al., 2026; Thomas-Rüddel et al., 2021); continuous formats require tens of minutes to equilibrate before the first reading (Wu et al., 2024)	Derived
Sampling medium	Whole blood	The only compartment with established reference ranges, kinetics and outcome associations (Koskela et al., 2017; Section 2.3); avoids centrifugation and preserves the point-of-care advantage	Established
Measurement mode	Single use	Biofouling restricts blood-based measurement to single-use formats (Liu et al., 2019; Saxena et al., 2024); endpoint operation on a freshly prepared electrode limits drift (Pham et al., 2025)	Derived
Multiplexing	4–5 channels	No single mediator captures the heterogeneity of the septic response; panels and inter-mediator ratios carry information that individual analytes do not (Tanak et al., 2022; Wang et al., 2026)	Extrapolated
Calibration stability	Preserved across measurements in the same patient	Decision rules in critical care are built on relative change from a prior value rather than on absolute thresholds (de Jong et al., 2016); comparable data for cytokine sensors are not available	Extrapolated
Antifouling strategy	Required	Blood carries the highest protein load and the most adhesive cellular components among candidate media (Sabaté del Río et al., 2019; Jiang et al., 2020)	Established
Cost per test	Cost per result lower than laboratory immunoassay (ELISA/CLIA)	The burden of sepsis falls disproportionately on lower-resource health systems (Li et al., 2026); aptamer receptors are cheaper to produce and more readily scaled (Crivianu-Gaita and Thompson, 2016)	Extrapolated

Evidence: Established - the requirement follows from measured concentrations or from demonstrated device performance; Derived - the requirement follows from an established clinical rule or guideline recommendation; Extrapolated - the requirement is a design judgment for which validating data in cytokine biosensing are not yet available. A limit of detection of 5 pg/mL does not resolve physiological concentrations of IL-1β or the lower end of the TNF-α reference range; this is not a limitation for the intended use, since the device is required to quantify deviation in a septic patient rather than to establish reference values.

Similarly, while the idea of continuous monitoring is well suited to “everyday” prevention and the management of chronic conditions (Tegegne et al., 2026), in the context of sepsis it may turn out to be more problematic than beneficial. In the case of ISF, there is a delay of several tens of minutes between connecting the sensor for continuous monitoring and obtaining the first stable reading (Wu et al., 2024). In this type of biosensing, local production of pro-inflammatory cytokines may also occur as a response to sensor implantation (Carnicer-Lombarte et al., 2021; Wu et al., 2024). Furthermore, as endothelial barrier disruption progresses, one can assume increasingly intense fouling of the electrode surface. When designing sensors, one should also deliberately prioritize a wider linear range and shorter measurement time over maximal analytical accuracy. As shown in Table 1, cytokine concentrations in sepsis can vary by several orders of magnitude, and sepsis itself rarely develops in patients whose cytokine levels are within physiological limits. From a clinical perspective, therefore, what matters are not changes of a few pg/mL or fractions thereof but a change spanning one or more orders of magnitude. The extent to which reported devices meet these requirements can be assessed directly. Of the 25 electrochemical cytokine biosensors compiled in Supplementary Table S1, two combine a linear range spanning at least three orders of magnitude with a response time under ten minutes, and two were evaluated in whole blood. Several report ranges lying entirely below or entirely above the concentrations encountered in sepsi.

### 4.2 Novelty and future scope

Most current work on cytokine biosensing is organized around a technological ambition: continuous, minimally invasive, wearable measurement (Ploner et al., 2024; Duan et al., 2025). The contribution of this Perspective is to invert that starting point and derive sensor specifications from the pathophysiology and workflow of the septic patient in intensive care. Doing so yields a different set of priorities - a wide dynamic range rather than the lowest attainable limit of detection, response times compatible with resuscitation timelines, blood as the sampling medium, and single-use operation - several of which run counter to the direction the field is currently taking.

For the most common target cytokines, antibody-, aptamer- and MIP-based platforms can all reach adequate analytical performance, so the decisive factors shift toward cost, scalability and matrix compatibility. Because the burden of sepsis falls disproportionately on lower-resource health systems, affordability is itself a design requirement. On this criterion, aptamers currently appear the most attractive option: cheaper to produce, longer-lived, readily modified with thiol groups or redox reporters, and composed of nucleic acids that interact predictably with biological matrices. Endpoint measurement reinforces this advantage, since brief exposure of the receptor layer relaxes the requirements for long-term stability (Crivianu-Gaita and Thompson, 2016; Li et al., 2026). For those cytokines for which aptamers are not yet available, efforts should be directed at generating them.

This conclusion is, however, provisional in an important respect. The choice between sampling media rests on a thin evidentiary base: information on cytokine concentrations across various compartments in septic patients is limited in scope (Koskela et al., 2017), and transfer across a barrier that sepsis itself disrupts (Marx, 2003) has been inferred rather than measured. The rate of equilibration between blood and interstitial fluid, and the extent to which peripheral compartments track systemic concentrations during capillary leak, therefore remain open questions - a gap the current guidelines echo from the clinical side, judging the evidence insufficient to recommend novel rapid host-response diagnostics (Prescott et al., 2026). Until these gaps are addressed, the case for blood rests as much on the absence of validation elsewhere as on its intrinsic advantages. For now, because the entire body of knowledge on cytokine concentrations in sepsis rests on blood as the reference medium, blood should remain the dominant medium in sensor design.

Markers of this kind can genuinely support bedside therapeutic decisions. Experimental strategies include personalized immunomodulation with immunosuppressive or immunostimulatory agents, aimed at modulating the patient’s immune response and thereby preventing, or limiting the consequences of, organ failure driven by inflammation (Leventogiannis et al., 2022; Giamarellos-Bourboulis et al., 2026), an approach that has also been explored in refractory pediatric septic shock (Lee et al., 2025). Similarly, such devices may support more precise use of hemofiltration systems, enabling clinicians to target a narrow therapeutic window in which cytokine removal genuinely contributes to clinical improvement while at the same time avoiding the induction of immunosuppression (Waalders et al., 2025).

## 5 Conclusion

Just as in sepsis itself, the “one size fits all” paradigm has failed; a single type of biosensor cannot be expected to match all diseases in which cytokines play a role. In this Perspective, we have outlined what we consider to be the key requirements for an electrochemical biosensor intended for the diagnosis and monitoring of sepsis. These include, above all, blood-based measurement, a wide linear detection range, and the shortest possible incubation time. Although these requirements are not fully aligned with the prevailing paradigms of biosensor design, they may prove crucial for the efficient quantification of cytokines in sepsis. In our view, effective use of existing biosensor fabrication strategies, combined with consciously tailoring them to the specific demands of sepsis, can substantially facilitate differential diagnosis and subsequent monitoring of the patient’s status during therapy. It must be remembered, however, that sensor research does not develop in a vacuum: sound design and subsequent clinical use also require basic physiological research and validation of these devices under real hospital conditions.

## Data Availability

The original contributions presented in the study are included in the article/Supplementary Material, further inquiries can be directed to the corresponding author.
