# Supplementary material for: Bridging sepsis physiology and biosensor engineering: a clinical perspective on electrochemical cytokine sensor design
Source: Front Bioeng Biotechnol. 2026 Sep 17;14:1942504. doi: 10.3389/fbioe.2026.1942504 (PMC13627097; doi:10.3389/fbioe.2026.1942504)
Supplement: Supplementary file 1 [file Table1.docx]

**Supplementary Table S1. Reported electrochemical biosensors for cytokines relevant to sepsis.**

Devices were included only if they target at least one cytokine listed in Table 1, with priority given to reports providing both a limit of detection and a linear range; the table is illustrative rather than exhaustive. Where a device was the only one available for a given target (e.g. IP-10) or for an early-stage recognition strategy (e.g. molecularly imprinted polymers), it was included even if it was not characterized in a matrixdiscussed in this article. Analytical figures of merit are reported as published and cannot be assumed to transfer to septic patients: the concentration ranges, matrices, sampling formats and intended indications of these studies differ from those encountered in sepsis, and none of the listed devices has been validated for that indication. Where a device was calibrated in buffer and only subsequently confirmed in a biological fluid, both are given in the Matrix column, calibration first.

| **#** | **Target(s)** | **Recognition** | **Platform / materials** | **Technique** | **LOD (pg/mL)** | **Linear range (pg/mL)** | **Span** | **Matrix** | **Time** | **Mode** | **Reference** | **Notes** |
| --- | --- | --- | --- | --- | --- | --- | --- | --- | --- | --- | --- | --- |
| 1 | IL-6 | Ab (sandwich) | cAb-magnetic beads + biotinylated dAb/streptavidin-HRP; AuNP/rGO on miniaturized Au electrode (glass); PDMS microfluidic chamber | Amperometry (HRP/TMB label, −0.8 V) | 0.412 | 0.97–250 | 2.4 | PBS (calibration); spiked human serum (recovery 93.4–103.8%) | 15 min on-chip readout; > 12 h total assay | Single-use | Zhang et al., 2022 | Only labeled enzymatic (HRP/TMB) assay here and, being a sandwich format, the closest analogue to ELISA in this table. The 15 min is the on-chip substrate incubation only: the sandwich is built off-chip (overnight capture at 4 °C, 1 h detection antibody, 30 min streptavidin-HRP). The limit of detection is given as 0.412 pg/mL in the results, 0.42 in the abstract and 0.41 in the authors’ own comparison table. Signal 79.8% of day 1 after 14 d; interference < 10% from CCL2, IL-1, TNF-α and CRP at 250 pg/mL; spiked serum only, no patient cohort. |
| 2 | IL-6 | Ab (competitive) | Ab/ERGO-AuPdNP/heated carbon paste (49 °C) + IL-6–PS@PDA–AgNP label | LSV | 0.059 | 0.1–100,000 | 6.0 | PBS (calibration); patient serum (validation vs ELISA) | 40 min | Single-use | Lou et al., 2014 | Competitive format — signal decreases with rising IL-6. Six orders of magnitude achieved only with dual amplification (heated electrode + AgNP label); on unheated carbon paste the same assay gave 10–1000 pg/mL and LOD 2.45 pg/mL |
| 3 | IL-6, IL-8, IL-10, TRAIL, IP-10 | Ab (label-free) | mAb/ZnO/Au | Non-faradaic EIS | 0.1–1 (analyte-dependent; 0.1 for IL-6 and IL-8) | 0.01–10,000 (IL-6); others narrower (IL-8 0.1–5000; IL-10 0.1–1000; TRAIL 1–1000; IP-10 1–2000) | 6.0 (IL-6) | Human plasma (undiluted) | 5 min | Single-use | Tanak et al., 2021 | 5-plex sepsis panel; < 40 µL undiluted plasma; n = 40 (20 septic / 20 healthy); Pearson r ≥ 0.90 vs Luminex. Abstract quotes a pooled ‘~1 pg/mL’; the reported IL-10 LOD exceeds the stated lower limit of its own range |
| 4 | IL-6, IL-8, IL-10, IP-10, TRAIL, d-dimer, CRP, G-CSF | Ab (label-free) | Ab/ZnO/Au | EIS | NR | NR | — | Human whole blood (undiluted) | 5 min | Single-use | Tanak et al., 2022 | 8-plex; one of two devices in this table evaluated in undiluted whole blood (see #22). No LOD or linear range reported — only recovery 105 ± 6%, R² > 0.97 and diagnostic cut-offs. 100 µL (two drops); n = 40 (30 septic / 10 healthy); r > 0.87 vs Luminex; AUC = 1.0 for IL-6 and CRP; 80–1000 Hz |
| 5 | IL-6, IL-8, IL-10, TNF-α | Ab (label-free) | mAb/ZnO on absorbent patch | Non-faradaic EIS | NR (Kₔ 0.13–0.83 pM) | 0.2–200 | 3.0 | Human eccrine sweat (passive) | 1 h per reading | Wearable, continuous (hourly sampling) | Jagannath et al., 2021 | 4-plex, wireless reader; r = 0.99 vs ELISA (n = 26); sensor surface not regenerated |
| 6 | IL-6 | Ab (label-free) | mAb/3-MPA/Au interdigitated array, BSA-blocked | DPV, EIS | 1.63 (PBS); 2.34 (CSF); 11.83 (serum) | 10–10,000 | 3.0 | Human CSF, serum | ~3 min (readout); 20–30 min incubation | Single-use | Oh et al., 2021 | R_ct decreased with IL-6 (opposite to expectation); ~10× selectivity over IL-10 and ~100× over TNF-α. The ‘3 min’ figure is the measurement step, not the assay |
| 7 | IL-6 | Ab (label-free) | Ab on 8 × 50 µm Au disc array, silicon needle substrate | DPV, EIS | 20 (lowest concentration tested; formal LOD not determined) | 0–60 | ~0.5 (nominal) | PBS (calibration); 5% BSA; human serum (single spiked point) | 2.5 min | Single-use | Russell et al., 2019 | Shortest incubation reported here; R_ct decreased with IL-6 on the microelectrodes but increased on macro screen-printed electrodes run in parallel |
| 8 | IL-6 | Aptamer | Apt/EG3 SAM/AuNP/Au | EIS | 0.02 (LOQ 0.05) | 0.02–20 | 3.0 | PBS (calibration); commercial artificial sweat (spiked) | NR | Single-use | Selva Kumar et al., 2016 | Response saturates above 20 pg/mL; 90% of signal after 2 weeks at 4 °C; single-use — reuse would require a dissociation step |
| 9 | IL-6 | Aptamer | Apt/AuNP/PPyNP/screen-printed graphite | EIS | 0.33 | 1–15,000,000 | 7.2 | PBS (calibration); commercial human serum 1:100 | 30 min (37 °C) | Single-use | Tertiş et al., 2017 | Widest reported range; complete sensor fabrication in ~30 min by multipulse amperometry |
| 10 | IL-6 | Aptamer | Apt/AuNP/pATP/pABA/GCE | EIS | 1.66 (LOQ 5) | 5–100,000 | 4.3 | Human serum diluted 1:1 | 60 min | Single-use | Tertis et al., 2019 | Three patients with colorectal cancer; validated against CLIA; 81% of signal after 28 days |
| 11 | IL-6, IL-8, TNF-α, TGF-β1 | Aptamer (MB-tagged) | Apt-MB/AuNP-graphene on polyurethane or PET | SWV (cytokines); OCP (pH) | NR | IL-6 0–30,000; IL-8 0–30,000; TNF-α 0–2000; TGF-β1 0–150 | — | Spiked serum (calibration); wound exudate (clinical) | 30 min | Continuous | Gao et al., 2021 | 4-plex; LOD not reported; calibrated in serum, applied to venous ulcer exudate |
| 12 | IL-6 | MIP | MIP-polypyrrole/carbon SPE | EIS | 0.02 (lowest calibration point; text states LOD < 0.1) | 0.1–20,000 | 5.3 | PBS (calibration); spiked human serum | 30 min | Single-use | Gonçalves et al., 2021 | R_ct trend opposite to other MIP sensors |
| 13 | IL-6 | MIP | MIP poly(3-aminophenylboronic acid) on Pt-metallized polycarbonate microneedle array | EIS (CV for characterization) | 1.0 | 1–10,000 | 4.0 | Artificial interstitial fluid only | 20 min incubation; 6 min measurement | Transdermal, single-use | Oliveira et al., 2022 | MIP described as ‘plastic antibody’; template removed with proteinase K; slope 0.69 Ω/decade; NIP control non-linear |
| 14 | IL-1β | MIP | poly(eriochrome black T)/PEDOT/4-AMP on flexible SPCE | EIS (CV for characterization) | 252 (LOQ 765) | 100–1000 | 1.0 | PBS only | 45–60 min | Single-use | Park et al., 2023 | LOD lies within the stated linear range and above both healthy and septic IL-1β values; NIP control also linear (R² 0.947 vs 0.973 for MIP); smartphone POCT with 3D-printed fluidic cartridge |
| 15 | IL-1β | MIP | poly(o-phenylenediamine)/poly(chromotrope 2R) on SPCE | EIS (CV, DPV) | 0.23 (LOQ 0.78) | 0.1–1050 | 4.0 | PBS (calibration, EIS); human serum 1:1000 (DPV and EIS) | 2 h binding | Single-use | Choi et al., 2022 | Same group as Park et al., 2023; binding time incompatible with acute care |
| 16 | IL-6 | Aptamer (ratiometric) | Electropolymerised methylene blue/chitosan-MWCNT on SPCE | DPV | 0.54 | 1–1,000,000 | 6.0 | Buffer (calibration); spiked human serum, 10× diluted | 30 min | Single-use | Liu et al., 2024 | Internal reference enables self-calibration; recovery 94.9–103.0%; 82.1% of signal after 14 d; inter-electrode RSD 3.7% |
| 17 | TNF-α | Aptamer | EGFET, monolayer graphene, rigid Cr/Au | FET (electrolyte-gated) | 10 | 10–1000 (tested to 10,000) | 2.0 | 1× PBS buffer | 15 min | Single-use chip (toward wearable) | Khorrami Jahromi et al., 2022 | Kₔ 21.74 pg/mL; range stated to represent sweat levels but never measured in sweat |
| 18 | TNF-α | Aptamer | FET, flexible/stretchable Cr/Au | FET | 86.5 (5 pM) | 865–1,730,000 (50 pM – 100 nM) | 3.3 | 1× PBS only | NR | Flexible/stretchable, toward wearable | Wang et al., 2019 | Kₔ 14.35 nM. TNF-α was measured in buffer only; the hand and contact-lens demonstrations show mechanical conformability, not biofluid sensing |
| 19 | TNF-α | Aptamer | FET, flexible Cr/Au, dual-channel | FET | 10.5 (611 fM) | 173–8,650,000 (0.01–500 nM) | 4.7 | 1× PBS (calibration); serum, saliva and urine (5–50 pM) and sweat (10–200 pM) on the PET version | 7 min | Wearable | Hao et al., 2021 | Widest range among TNF-α devices. Dual-channel design — reference channel with TWEEN 80 passivation compensating pH and ionic-strength drift — is the only such arrangement in this table; the platform also detects IL-6 (608 fM) and IFN-γ (476 fM) |
| 20 | TNF-α | Aptamer | Flexible carbon SPCE on PET/AuNP/thiolated aptamer, PEG-blocked + PDMS microfluidics | EIS (redox-free; \|Z\| at 10 Hz) | 3.2 (sweat); 0.64 (PBS) | 0.2–1000 | 3.7 | 1× PBS (calibration, static); artificial sweat under continuous flow (20 µL/min) | n/a (continuous flow) | Continuous | Ploner et al., 2025 | Only continuous-flow device in this table. LOD 0.64 pg/mL in static 1× PBS vs 3.2 pg/mL in flowing artificial sweat; sensitivity falls from 160.9 ± 8.9 to 101.96 ± 11.4 Ω/log(pg/mL) and the standard error rises from 26.8 to 167 Ω under flow. Stable to day 10, then a sharp decline. Selectivity rests on one test: 500 pg/mL IL-6 alone shifted the signal by 213 ± 64 Ω against 396 ± 47 Ω for the same solution plus 50 pg/mL TNF-α. Never tested in real human sweat; no aptamer regeneration. |
| 21 | IL-10, IFN-γ | Ab | Aerosol-jet-printed graphene-nitrocellulose IDE on polyimide | EIS | IFN-γ 25; IL-10 46 | IFN-γ 100–5000; IL-10 100–2000 | 1.7 / 1.3 | Bovine minced implant supernatant (serum), diluted 1:1000 | 30 min incubation; 33 min response | Single-use, flexible | Parate et al., 2020 | Mouse anti-bovine antibodies; ΔR_ct ~6% after 250 bending cycles |
| 22 | IL-10, IL-18 | Ab | READ platform; ZnO on Au, 16-electrode chip | Non-faradaic EIS | NR | IL-10 1–40; IL-18 170–717 | 1.6 / 0.6 | K₂-EDTA whole blood and plasma (≤160 µL) | < 10 min (7 min incubation) | Point-of-care | David et al., 2026 | Dual pro-/anti-inflammatory readout; recovery 80–120%, R² 0.98–0.99; higher variability and plateauing response in whole blood vs plasma |
| 23 | CXCL9, IP-10 (CXCL10) | Ab (label-free) | SPCE/Ti₃C₂Tₓ MXene-crosslinked BSA hydrogel | CV with [Ru(NH₃)₆]³⁺ | CXCL9 6.9; CXCL10 5.7 | 7–1000 (log-linear) | 2.2 | Unprocessed urine (5 µL) | 15 min | Single-use | Gupta et al., 2026 | Antifouling; stable ≥ 3 weeks (< 5% loss over 4 weeks); 60-patient cohort; classifier 83% → 98% with clinical features |
| 24 | IP-10 (CXCL10) | Peptide–PNA chimera (E-DNA scaffold) | Au disk electrode, methylene blue | SWV | NR (low nanomolar) | ~10,000–20,000,000 (1 nM – 2 µM titration) | 3.3 | Undiluted fetal calf serum | > 30 min equilibration | Single-use | Bonham et al., 2013 | Label-free, wash-free; authors state sensitivity falls short of the ~60 pM clinical level (peptide Kₔ ~200 nM); signal change ~30% in buffer, halved in serum |
| 25 | IL-10, IFN-γ | Ab (label-free) | Mouse anti-bovine Abs on vertically aligned CNT (VANTA) interdigitated electrodes on silicon; EDC/NHS | Faradaic EIS (ΔR_ct) | IL-10 0.0911; IFN-γ 24.17 | IL-10 0.1–30; IFN-γ 50–500 | 2.5 / 1.0 | 1× PBS (calibration); bovine implant supernatant diluted 1:1000 | < 35 min | Single-use | Ding et al., 2024 | Veterinary — stage-I Johne’s disease screening in cattle; rigid silicon substrate, two-electrode read-out. The article gives four different IL-10 limits of detection for the same matrix (0.077 in the introduction, 0.0911 in the abstract, 0.09112 in the results, 0.0848 in Figure 5 and the conclusion) and two upper limits for IFN-γ (500 vs 1000 pg/mL); the abstract values are used here. Response falls at the highest concentrations (receptor saturation); interferent testing included IL-6. |

**Footnotes.** Span is the base-10 logarithm of the ratio of the upper to the lower limit of the reported linear range; it is nominal where the lower limit of the reported range was zero but a limit of detection was available, in which case the latter was used as the lower bound, and is omitted where neither was reported. For entries 17–19 the limits of detection and linear ranges were converted from molar units assuming the TNF-α monomer (17.3 kDa); the biologically active trimer (52 kDa) would treble these figures. For entry 24 the conversion assumes IP-10 (10 kDa).

**Abbreviations.** 3-MPA, 3-mercaptopropionic acid; 4-AMP, 4-aminothiophenol; Ab, antibody; AgNP, silver nanoparticle; APBA, 3-aminophenylboronic acid; Apt, aptamer; AUC, area under the receiver-operating-characteristic curve; AuNP, gold nanoparticle; AuPdNP, gold–palladium nanoparticle; BSA, bovine serum albumin; CLIA, chemiluminescence immunoassay; CNT, carbon nanotube; CRP, C-reactive protein; CSF, cerebrospinal fluid; CV, cyclic voltammetry; CXCL9/CXCL10, C-X-C motif chemokine ligand 9/10; DPV, differential pulse voltammetry; EG3, tri(ethylene glycol)-terminated alkanethiol; EIS, electrochemical impedance spectroscopy; ELISA, enzyme-linked immunosorbent assay; ERGO, electrochemically reduced graphene oxide; FET, field-effect transistor; f-MWCNT, functionalised multi-walled carbon nanotube; G-CSF, granulocyte colony-stimulating factor; GCE, glassy carbon electrode; HRP, horseradish peroxidase; IDE, interdigitated electrode; IFN-γ, interferon gamma; IL, interleukin; IP-10, interferon-γ-inducible protein 10; ISF, interstitial fluid; ITO, indium tin oxide; Kₔ, dissociation constant; LOD, limit of detection; LOQ, limit of quantification; LSV, linear sweep voltammetry; mAb, monoclonal antibody; MB, methylene blue; MIP, molecularly imprinted polymer; MWCNT, multi-walled carbon nanotube; NIP, non-imprinted polymer; NR, not reported; OCP, open-circuit potentiometry; pAb, polyclonal antibody; pABA, para-aminobenzoic acid; pATP, p-aminothiophenol; PBS, phosphate-buffered saline; PDMS, polydimethylsiloxane; PEDOT, poly(3,4-ethylenedioxythiophene); PET, polyethylene terephthalate; PNA, peptide nucleic acid; POCT, point-of-care testing; PPy(NP), polypyrrole (nanoparticle); PS@PDA, polystyrene sphere coated with polydopamine; R_ct, charge transfer resistance; rGO, reduced graphene oxide; SPCE, screen-printed carbon electrode; SPE, screen-printed electrode; SWV, square-wave voltammetry; TGF-β1, transforming growth factor beta 1; TNF-α, tumour necrosis factor alpha; TRAIL, TNF-related apoptosis-inducing ligand.

**References cited in Supplementary Table S1**

Bonham, A. J., Paden, N. G., Ricci, F., and Plaxco, K. W. (2013). Detection of IP-10 protein marker in undiluted blood serum via an electrochemical E-DNA scaffold sensor. Analyst 138, 5580–5583. doi: 10.1039/c3an01079a

Choi, D. Y., Yang, J. C., Hong, S. W., and Park, J. (2022). Molecularly imprinted polymer-based electrochemical impedimetric sensors on screen-printed carbon electrodes for the detection of trace cytokine IL-1β. Biosens. Bioelectron. 204, 114073. doi: 10.1016/j.bios.2022.114073

David, B. E., Madhurantakam, S., Mathew, G., Dhamu, V. N., Parulekar, S., Krovvidi, A. S., et al. (2026). Dual-cytokine profiling of inflammatory states using a rapid electroanalytical device as a point-of-care sensor platform. Microchim. Acta 193, 167. doi: 10.1007/s00604-026-07886-y

Ding, S., Brownlee, B. J., Parate, K., Pola, C. C., Chen, B., Hostetter, J. M., et al. (2024). IFN-γ and IL-10 immunosensor with vertically aligned carbon nanotube interdigitated electrodes toward pen-side cattle paratuberculosis monitoring. Global Challenges 8, 2400021. doi: 10.1002/gch2.202400021

Gao, Y., Nguyen, D. T., Yeo, T., Lim, S. B., Tan, W. X., Madden, L. E., et al. (2021). A flexible multiplexed immunosensor for point-of-care in situ wound monitoring. Sci. Adv. 7, eabg9614. doi: 10.1126/sciadv.abg9614

Gonçalves, M. de L., Truta, L. A. N., Sales, M. G. F., and Moreira, F. T. C. (2021). Electrochemical point-of-care determination of interleukin-6 (IL-6) using a pyrrole (Py) molecularly imprinted polymer (MIP) on a carbon-screen printed electrode (C-SPE). Anal. Lett. 54, 2611–2623. doi: 10.1080/00032719.2021.1879108

Gupta, R., Salaris, N., Kalkal, A., Chang, F. Y., Javed, M., Khan, A. A., et al. (2026). Electrochemical biosensor for rapid detection of acute rejection in kidney transplants. Adv. Healthc. Mater. 15, e02831. doi: 10.1002/adhm.202502831

Hao, Z., Luo, Y., Huang, C., Wang, Z., Song, G., Pan, Y., et al. (2021). An intelligent graphene-based biosensing device for cytokine storm syndrome biomarkers detection in human biofluids. Small 17, 2101508. doi: 10.1002/smll.202101508

Jagannath, B., Lin, K.-C., Pali, M., Sankhala, D., Muthukumar, S., and Prasad, S. (2021). Temporal profiling of cytokines in passively expressed sweat for detection of infection using wearable device. Bioeng. Transl. Med. 6, e10220. doi: 10.1002/btm2.10220

Khorrami Jahromi, A., Shieh, H., Low, K., Tasnim, N., Najjaran, H., and Hoorfar, M. (2022). Experimental comparison of direct and indirect aptamer-based biochemical functionalization of electrolyte-gated graphene field-effect transistors for biosensing applications. Anal. Chim. Acta 1222, 340177. doi: 10.1016/j.aca.2022.340177

Liu, Z., Liu, F., Wang, C., Li, H., Xu, Y., and Sun, S. (2024). Ratiometric electrochemical detection of interleukin-6 using electropolymerized methylene blue and a multi-walled carbon-nanotube-modified screen-printed carbon electrode. Biosensors 14, 457. doi: 10.3390/bios14100457

Lou, Y., He, T., Jiang, F., Shi, J.-J., and Zhu, J.-J. (2014). A competitive electrochemical immunosensor for the detection of human interleukin-6 based on the electrically heated carbon electrode and silver nanoparticles functionalized labels. Talanta 122, 135–139. doi: 10.1016/j.talanta.2014.01.016

Oh, C., Park, B., Li, C., Maldarelli, C., Schaefer, J. L., Datta-Chaudhuri, T., et al. (2021). Electrochemical immunosensing of interleukin-6 in human cerebrospinal fluid and human serum as an early biomarker for traumatic brain injury. ACS Meas. Sci. Au 1, 65–73. doi: 10.1021/acsmeasuresciau.1c00013

Oliveira, D., Correia, B. P., Sharma, S., and Moreira, F. T. C. (2022). Molecular imprinted polymers on microneedle arrays for point of care transdermal sampling and sensing of inflammatory biomarkers. ACS Omega 7, 39039–39044. doi: 10.1021/acsomega.2c04789

Parate, K., Rangnekar, S. V., Jing, D., Mendivelso-Perez, D. L., Ding, S., Secor, E. B., et al. (2020). Aerosol-jet-printed graphene immunosensor for label-free cytokine monitoring in serum. ACS Appl. Mater. Interfaces 12, 8592–8603. doi: 10.1021/acsami.9b22183

Park, R., Jeon, S., Lee, J. W., Jeong, J., Kwon, Y. W., Kim, S. H., et al. (2023). Mobile point-of-care device using molecularly imprinted polymer-based chemosensors targeting interleukin-1β biomarker. Biosensors 13, 1013. doi: 10.3390/bios13121013

Ploner, M., Shkodra, B., Franchin, L., Altana, A., Petrelli, M., Costa Angeli, M. A., et al. (2025). Flexible microfluidics-integrated electrochemical system for detection of tumor necrosis factor-alpha under continuous flow of sweat. Biosens. Bioelectron. 287, 117734. doi: 10.1016/j.bios.2025.117734

Russell, C., Ward, A. C., Vezza, V., Hoskisson, P., Alcorn, D., Steenson, D. P., et al. (2019). Development of a needle shaped microelectrode for electrochemical detection of the sepsis biomarker interleukin-6 (IL-6) in real time. Biosens. Bioelectron. 126, 806–814. doi: 10.1016/j.bios.2018.11.053

Selva Kumar, L. S., Wang, X., Hagen, J., Naik, R., Papautsky, I., and Heikenfeld, J. (2016). Label free nano-aptasensor for interleukin-6 in protein-dilute bio fluids such as sweat. Anal. Methods 8, 3440–3444. doi: 10.1039/C6AY00331A

Tanak, A. S., Muthukumar, S., Krishnan, S., Schully, K. L., Clark, D. V., and Prasad, S. (2021). Multiplexed cytokine detection using electrochemical point-of-care sensing device towards rapid sepsis endotyping. Biosens. Bioelectron. 171, 112726. doi: 10.1016/j.bios.2020.112726

Tanak, A. S., Sardesai, A., Muthukumar, S., and Prasad, S. (2022). Simultaneous detection of sepsis host response biomarkers in whole blood using electrochemical biosensor. Bioeng. Transl. Med. 7, e10310. doi: 10.1002/btm2.10310

Tertiş, M., Ciui, B., Suciu, M., Săndulescu, R., and Cristea, C. (2017). Label-free electrochemical aptasensor based on gold and polypyrrole nanoparticles for interleukin 6 detection. Electrochim. Acta 258, 1208–1218. doi: 10.1016/j.electacta.2017.11.176

Tertis, M., Leva, P. I., Bogdan, D., Suciu, M., Graur, F., and Cristea, C. (2019). Impedimetric aptasensor for the label-free and selective detection of interleukin-6 for colorectal cancer screening. Biosens. Bioelectron. 137, 123–132. doi: 10.1016/j.bios.2019.05.012

Wang, Z., Hao, Z., Yu, S., De Moraes, C. G., Suh, L. H., Zhao, X., et al. (2019). An ultraflexible and stretchable aptameric graphene nanosensor for biomarker detection and monitoring. Adv. Funct. Mater. 29, 1905202. doi: 10.1002/adfm.201905202

Zhang, C., Shi, D., Li, X., and Yuan, J. (2022). Microfluidic electrochemical magnetoimmunosensor for ultrasensitive detection of interleukin-6 based on hybrid of AuNPs and graphene. Talanta 240, 123173. doi: 10.1016/j.talanta.2021.123173
